# Assessing plant community composition fails to capture impacts of white-tailed deer on native and invasive plant species

**DOI:** 10.1093/aobpla/plx026

**Published:** 2017-06-08

**Authors:** Victoria Nuzzo, Andrea Dávalos, Bernd Blossey

**Affiliations:** 1 Natural Area Consultants, 1 West Hill School Road, Richford, NY 13835, USA; 2 Department of Natural Resources, 206 Fernow Hall, Cornell University, Ithaca, NY 14853, USA

**Keywords:** Deer herbivory, deer management, earthworms, forest understory, invasive species, multiple stressors, plant community

## Abstract

Excessive herbivory can have transformative effects on forest understory vegetation, converting diverse communities into depauperate ones, often with increased abundance of non-native plants. White-tailed deer are a problematic herbivore throughout much of eastern North America and alter forest understory community structure. Reducing (by culling) or eliminating (by fencing) deer herbivory is expected to return understory vegetation to a previously diverse condition. We examined this assumption from 1992 to 2006 at Fermilab (Batavia, IL) where a cull reduced white-tailed deer (*Odocoileus virginianus*) abundance in 1998/1999 by 90 % from 24.6 to 2.5/km^2^, and at West Point, NY, where we assessed interactive effects of deer, earthworms, and invasive plants using 30 × 30 m paired fenced and open plots in 12 different forests from 2009 to 2012. We recorded not only plant community responses (species presence and cover) within 1 m^2^ quadrats, but also responses of select individual species (growth, reproduction). At Fermilab, introduced *Alliaria petiolata* abundance initially increased as deer density increased, but then declined after deer reduction. The understory community responded to the deer cull by increased cover, species richness and height, and community composition changed but was dominated by early successional native forbs. At West Point plant community composition was affected by introduced earthworm density but not deer exclusion. Native plant cover increased and non-native plant cover decreased in fenced plots, thus keeping overall plant cover similar. At both sites native forb cover increased in response to deer reduction, but the anticipated response of understory vegetation failed to materialize at the community level. Deer-favoured forbs (*Eurybia divaricata*, *Maianthemum racemosum*, *Polygonatum pubescens* and *Trillium recurvatum*) grew taller and flowering probability increased in the absence of deer. Plant community monitoring fails to capture initial and subtle effects of reduced or even cessation of deer browse on browse sensitive species. Measuring responses of individual plants (growth, flowering and reproductive success) provides a more sensitive and powerful assessment of forest understory responses to deer management.

## Teaser

Excessive herbivory by native deer can reduce abundance of native vegetation and increase non-native vegetation, but how does the forest recover when deer are excluded or numbers are reduced? The expectation is that understory vegetation will return to a previously diverse condition, but this is not always the case. We found that community-level monitoring captured both change and lack of change in vegetation cover, height, species richness, and response of common species, but failed to capture response of uncommon species. In contrast, monitoring of target species favored by deer indicated increased plant size and reproduction when deer were reduced or excluded. We conclude that measuring individual plants (growth, flowering and reproductive success) provides a sensitive and powerful assessment of forest understory responses to deer management.

## Introduction

Conservation successes should be celebrated, but the acclaim is muted when recovery of a species is so successful that its abundance threatens other species. Adaptable native and introduced wild or feral species (including browsers and grazers such as deer and kangaroos) thrive in landscapes transformed by humans over the past two centuries in Europe, Australia, Japan, New Zealand and North America (Forsyth *et al.* 2010; Tanentzap *et al.* 2012; Foster *et al.* 2014; Howland *et al.* 2014; Perea *et al.* 2014; Iijima and Ueno 2016).

The success of white-tailed deer (*Odocoileus virginianus*) in North America illustrates a conservation success gone awry. Hunted to near extinction by the late 1800s, the species made such a remarkable comeback that large populations quickly created economic and ecological problems (Leopold *et al.* 1947; Severinghouse and Brown 1956; Halls 1984). Scientists initially focused on selective effects of deer, primarily on valuable timber species (Habeck 1959; Frelich and Lorimer 1985; Horsley *et al.* 2003). While some browsing can have beneficial effects on plant diversity by reducing the competitive ability of some fast growing species (Leonardsson *et al.* 2015), large ungulate populations often create simplified and less diverse ecosystems.

In the past decades, ecologists have documented cascading negative impacts of large deer populations (Porter and Underwood 1999; McShea and Rappole 2000; Côté *et al.* 2004; Griggs *et al.* 2006). These effects percolate through food webs affecting directly the plant species eaten, and indirectly species such as insects, birds and decomposers, that are dependent upon the diversity of primary producers for their own existence and livelihoods (Wardle and Bardgett 2004; McGraw and Furedi 2005; Martin *et al.* 2011; Nuttle *et al.* 2011; Schweitzer *et al.* 2014; Chips *et al.* 2015). This is not necessarily a new phenomenon because problems in Europe and North America have been reported for nearly a century or longer (Leopold *et al.* 1947; Schabel 2001; Ammer *et al.* 2010).

The complexities and interconnectedness of local and regional ecosystems, as well as the many potential drivers of change (climate, invasive species, trophic downgrading, etc.) (Tylianakis *et al.* 2008; Estes *et al.* 2011; Craven *et al.* 2016), obscure the current and future implications of ungulate impacts. Maintaining ecosystem function and ecosystem services requires high species diversity in multiple trophic groups including primary producers, above-ground insect herbivores and soil decomposers (Soliveres *et al.* 2016; van der Plas *et al.* 2016), organisms that are particularly negatively affected by high ungulate browse pressure (Côté *et al.* 2004).

Around the world foresters and managers have responded to high ungulate browse pressure by culling ungulate populations, or by fencing endangered plant populations and conservation areas. These are stopgap measures that can provide (at least in the case of trees) a temporary reprieve until increased height ensures survival of individuals. No such spatial escape exists for the vast majority of herbaceous plants that represent the staple of deer diets in spring and summer, and that are under particular threat (Williams *et al.* 2000; Fletcher *et al.* 2001; McGraw and Furedi 2005; Wiegmann and Waller 2006; Wang and Mopper 2008; Knight *et al.* 2009a; Frerker *et al.* 2014).

It is widely assumed that reduction or cessation of high deer browse pressure will result in the recovery of understory plant communities but field tests of these assumptions are rare (Tanentzap *et al.* 2011). When hunting, culling and fencing are used to address excessive deer browse, managers typically, at least in hunting and culling efforts, report the number of deer removed or the resulting deer population density (DeNicola 2008) (but see Wright *et al.* 2012). However, the extent to which vegetation recovery occurs is less often measured. Furthermore, composition and abundance of understory vegetation are not necessarily linked to changes in deer abundance (Tanentzap *et al.* 2011).

An important concern is the long-term impact or legacy effects of high deer populations that act in concert with other stressors on local species assemblages and ecosystems. This question has received less attention, although indirect or non-consumptive effects of white-tailed deer, including effects on introduced invasive plants or earthworms, are now being recognized (Knight *et al.* 2009b; Heckel *et al.* 2010; Kalisz *et al.* 2014; Dávalos *et al.*2015a, b, c). Such perturbations may shift ecoystems into alternative stable states where restoration of a state resembling pre-perturbation conditions may be difficult or impossible (Beisner *et al.* 2003; Suding *et al.* 2004; Suding 2011). Persistent and excessive deer herbivory may create such a regime shift by simplifying primary producer communities setting forests on depauperate long-term trajectories (Nuttle *et al.* 2011, 2014) and affecting ecosystem processes, such as decomposition (Wardle and Bardgett 2004) and microbial community composition, that in turn affect plant growth and potentially net primary productivity (Kardol *et al.* 2014)

We were particularly interested to assess the response of herbaceous understory vegetation to deer management in the presence of other persistent stressors, such as introduced plants and non-native earthworm invasions. We used a 90 % reduction in the deer herd at Fermilab, IL, USA and an exclosure network at West Point, NY, USA, to test the following hypotheses:

1. Reduced deer browse pressure will result in recovery of forest understory plant community.
2. Reduced deer browse pressure will result in recovery of species highly favoured by deer.
3. Deer browse pressure overwhelms negative effects of other co-occurring stressors, specifically non-native plants and non-native earthworms, on native plant species performance

## Methods

### Fermilab

#### Site

U.S. DOE Fermilab National Environmental Research Park (hereafter Fermilab) is located 60 km west of Chicago, in Kane County, IL, USA and contains a mixture of forests, oldfields, restored prairies and active farm lands on 27.5 km^2^. We selected the largest forest on the site, a 31.6-ha mesic forest (“Big Woods”) dominated by *Tilia americana, Fraxinus americana, Quercus macrocarpa* and *Q. alba*, and known for extensive spring displays of *Trillium flexipes* and *T. recurvatum*. The forest had been grazed some time prior to establishment of the Research Park. A rapidly expanding deer herd and concomitant decline in *Trillium* floral displays prompted research to assess deer impacts on understory vegetation. White-tailed deer densities averaged 11/km^2^ in 1993, and 24.6/km^2^ in 1998, according to winter aerial surveys. Deer densities were reduced ∼90 % in winter 1998/1999 and were subsequently maintained using sharpshooters at approximately the same density (2.5–6/km^2^) each year thereafter.

#### Vegetation data

*Community:* In 1992 we divided the forest into five 90-m wide units, each with a randomly established permanent transect (420–560 m). In May of even-numbered years (1992–2006) we recorded species presence, and estimated percent cover for each species <1 m tall in 17 cover categories (midpoints: 0.01, 0.2, 0.5, 1, 3, 5, 10, 20, 30, 40, 50, 60, 70, 80, 90, 98 and 100 %) in 1 m^2^ quadrats randomly located within contiguous 20 m intervals along each transect (121–129 quadrats each year). Plant species nomenclature and native/non-native origin follows the USDA Plants Database (USDA NRCS 2017). Beginning in 1998 we measured “average” vegetation height to the nearest cm. While this is a quantitative measurement of a qualitative feature, all measurements were made in a consistent manner, and gross differences are assumed to be indicative of true height differences. In 2004 and 2006 we recorded species presence and height in all quadrats, and percent cover of individual species in alternate quadrats (69 and 65, respectively). We summed cover of all species within a quadrat using cover class midpoint values: thus, total cover could exceed 100 % due to layering. We classified each species by origin (native or non-native) and life form (annual and biennial forb combined, hereafter referred to as (bi)annual, perennial forb, graminoid, and woody). Graminoids included all grass-like plants (Poaceae, Cyperaceae and Juncacaeae). Ferns were so infrequent that they were combined with perennial forbs.

*Individual species*: Beginning in May 1998 and prior to the initial deer cull we collected demographic data for *T. recurvatum* to supplement frequency and cover data. We recorded stem density, stem height (cm; measured to the junction of the three leaves or to the leaf base), life form (sterile, fertile, one leaf), and presence of browse on each stem within each quadrat.

### West Point

#### Site

US Army Garrison West Point (hereafter West Point) is a 65-km^2^ site located on the west bank of the Hudson River some 80 km north of New York City, NY, USA. This facility is located in the Hudson Highlands Province, a landscape characterized by steep hills, thin soils and rocky outcrops. We selected 12 upland deciduous forests (1–8 km apart from each other) dominated by oak (*Q. rubra* and *Q. prinus*) and/or sugar maple (*Acer saccharum*); sites differed in land use history, aspect, soil and understory vegetation. Our investigations focused, in part, on the importance of non-native invasive plants, non-native invasive earthworms and their individual, additive or synergistic interactions with white-tailed deer and the resulting impacts on native plant species. We therefore selected 12 sites based on site invasion: 6 sites based on presence and abundance of 3 non-native plant species (*Alliaria petiolata*, *Berberis thunbergii, Microstegium vimineum*; two sites each) and six sites that had few or no non-native plant species present (please see Table 1 in Dávalos *et al.* 2015c). At each site we established two paired 30 m × 30 m plots, 5–50 m apart, with similar overstory and understory composition of which one was randomly selected to be fenced. In early July 2008 we constructed deer-proof 2.6 m high fences (Millennium plastic deer fence, deerBusters.com).

**Table 1.** Effects of study year, plant species origin (O; native, non-native), earthworm density (E; low, high) and fencing (F; open, fenced) on vegetation cover (%) at 12 sites at West Point, NY from 2009 to 2012 according to linear mixed-model. Model included site, plot within site and quadrat within plot as random factors. Non-significant study factors were dropped from selected models and not included in the table.

Factor	Cover			
	Estimate*	SE*	*χ*^2^ (df = 1)**	P**
Intercept	0.046	0.008		
Year	0.002	0.001		
Origin (non-native)	−0.035	0.003		
Earthworm (high)	4.69E−04	0.009		
Fencing (open)	−0.008	0.002		
O × Y	−0.003	0.001	7.35	0.01
O × E	0.020	0.003	59.42	<0.001
O × F	0.014	0.002	33.05	<0.001

Est Estimate; SE estimate standard error.

* Estimates and standard errors are reported from the model fitted with restricted maximum likelihood.

** Chi-squared statistics and P-values are from likelihood ratio tests with each parameter removed from the maximum likelihood-based model, with all other parameters retained. It was not possible to test the significance of all terms because of higher order interactions.

#### Vegetation data

*Community:* We established ten 1 m^2^ permanent quadrats in a stratified random manner in each fenced and open plot in July 2008 (11 sites) or May 2009 (1 site) (12 sites × 2 plots per site × 10 quadrats per plot = 240 vegetation quadrats). Within each quadrat we recorded species presence, and estimated the percent cover for each species <1 m tall in 17 cover categories (midpoints: 0.01, 0.2, 0.5, 1, 3, 5, 10, 20, 30, 40, 50, 60, 70, 80, 90, 98 and 100 %). Plant species nomenclature and native/non-native status follows USDA Plants (USDA NRCS 2017). We recorded data twice a year, in mid-May (2009–2012) and late July (2008–2012), and again in May 2016. We estimated vegetation height by measuring the “average” height of vegetation at four locations within each quadrat, and then averaging these heights for each quadrat. We classified each species by origin and life form, and summed cover of individual plant species as described above under Fermilab.

*Individual species*: We selected three native perennial plant species favoured by deer (*Eurybia divaricata*, *Maianthemum racemosum* and *Polygonatum pubescens*) in 2012 for additional study. We assessed all sites for presence of each species but collected data only at sites where we could find a minimum number of individuals in both open and fenced plots (2 sites for *M. racemosum* and *P. pubescens* [25 plants/plot minimum] and 6 sites for *E. divaricata* [50 plants/plot minimum]).

In mid-June 2012 we randomly located four 2 m × 30 m belt transects in each fenced plot at sites 5 and 8 and recorded stem height (cm, measured from ground to highest leaf node), width (cm) of the widest leaf, and reproductive status of each *M. racemosum* and *P. pubescens*. We found very few (or no) plants in belt transects in open plots; therefore we randomly established additional transects near open plots for a total of eight belt transects at site 5 in 2012 (seven transects in 2016) and nine transects at site 8. We repeated these measures in approximately the same locations in late May 2016.

In mid-September 2012 we recorded density, height, and reproductive status of each *E. divaricata* in the 10 permanent 1 m^2^ quadrats in each open and fenced plot at the six sites that contained this species. If *E. divaricata* was not present in the initial quadrat location, we searched for the closest *E. divaricata*, and then placed the quadrat with the plant in one corner and shifted to maximize number of plants in the re-established quadrat. Thus, we recorded frequency and cover in initial quadrats, and plant vigor in initial and additional quadrats.

#### Earthworm monitoring

In late July 2008–2011 we randomly selected 5 of the 10 vegetation quadrats within each open and fenced plot, stratified equally across the plot, and sampled earthworms within a 0.25-m^2^ frame placed 1 m distant from the vegetation quadrat. To ensure equal sampling of plots over time, we rerandomized sample locations each year. We removed and sifted all leaf litter, capturing any earthworms, then firmly placed a metal frame (10 cm × 25 cm × 25 cm) on the ground. We then poured 3.79 L of aqueous mustard solution (15 g L^−^^1^; Frontier Natural Products Co-op, Norway, IA) evenly across the frame area, and collected all surfacing earthworms. We preserved specimens in 10 % formalin before transferring to 70 % ethanol, then weighed and identified each individual to species (sexually mature worms) or genus (immature worms). Sites varied in earthworm abundance and species composition, naturally grouping into either ‘high’ (12.4 ± 1.78 individuals and 8.6 ± 1.67 g per 0.25 m^2^; means ± 1 SE: eight sites) or ‘low’ (1.4 ± 0.37 individuals and 0.88 ± 0.36 g per 0.25 m^2^; means ± 1 SE: four sites) earthworm abundance based on means from 2008 to 2011 (Dávalos *et al.* 2015c). We only recorded non-native earthworm species. Common genera included *Amynthas, Dendrobaena* and *Lumbricus.*

### Statistical Analyses

We evaluated effect of deer reduction on understory plant communities at Fermilab by comparing cover, species richness and vegetation height values pre (before 1998) and post deer reduction [a fixed factor with two levels (before and after): GLMMs with study year as a random factor to account for temporal correlation within years]. Samples within Fermilab (all at one site) were considered independent. At both Fermilab and West Point we classified each species by origin (native or non-native) and life-form (annual and biennial forb, perennial forb, graminoid [Poaceae, Cyperaceae and Juncacaeae], ferns and fern allies, and woody). Also for both studies, we transformed cover values (arcsine squared-root) and species richness (log) in order to meet model assumptions.

West Point analyses are based on maximum species cover between May and July for each sampling year (2009–2012). We used Linear Mixed Models (LMM) to evaluate effects of year, site invasion (sites selected based on presence and abundance of three non-native plant species), fencing (fenced or open), earthworm density (low or high based on earthworm monitoring results) and their interactions on total cover, species richness and diversity indexes. We dropped non-significant factors from the final model. We tested second level interactions only, excluding the site invasion × earthworm density interaction as we only had one site dominated by non-native vegetation with low earthworm density. We applied a second set of LMM models to evaluate effects of year, fencing, earthworm density, plant species origin, and their interactions on total cover and cover by life form (different models for each life form). We did not evaluate site invasion in models that separated species cover by lifeform as site invasion and plant species origin are not independent: Sites classified as “invaded” will, by definition, have higher cover of non-natives. All models included site, plot within site and quadrat within plot as random factors in order to reflect the hierarchical nature of the data. Including random factors allowed us to control for possible pseudoreplication while preserving the spatial variation contained in data collected among quadrats within the same plot (Millar and Anderson 2004; Schank and Koehnle 2009).

To evaluate significance of explanatory factors, we started with a full model and then compared it with a model without the tested factor via a log likelihood test. All chi-square statistics and associated *P*-values in the results section refer to log likelihood test results. We checked assumptions of all models at each step of the model procedure. We fitted all mixed models with package lme4 (Bates *et al.* 2014) in statistical package R (R Core Team 2016).

We assessed plant community composition at both Fermilab and West Point with non-metric multidimensional scaling (NMDS) and permutational multivariate analysis of variance (PerMANOVA) including site and plot within site as strata (Anderson 2001). We fitted NMDS and PerMANOVA with the metaMDS and adonis functions, respectively, in R package vegan (Oksanen *et al.* 2012). We used Bray-Curtis distance matrices, based on log-transformed species abundance data for both procedures. We fitted our study factors [deer exclusion (West Point) or reduction (Fermilab), and earthworm abundance and site invasion (at West Point only)] to the ordination (NMDS) using the envirofit function, which provides a goodness of fit and p-value via a permutation procedure. We only included species with maximum cover higher than 0.01 % and present at least 10 times over the study period.

We conducted separate analyses for May 2016 data and did not evaluate temporal effects because we excluded two sites due to fence damage. We evaluated effects of study factors on percent cover with ANOVA using median vegetation cover across quadrats within a plot (*N* = 10) as response.

We used Linear Mixed Models to evaluate effects on individual species cover and height and Generalized Linear Mixed Models with binomial errors to evaluate effects on species frequency or flowering probability. *Trillium recurvatum* models at Fermilab evaluated changes before and after deer reduction (a fixed factor with two levels [before and after]) and included sampling year as a random factor to account for temporal correlation within years. Models for West Point evaluated the effects of fencing, earthworm density and site invasion and included site and plot within site as random factors.

## Results

### Fermilab

#### Community

##### Cover and vegetation height

**Figure 1.**
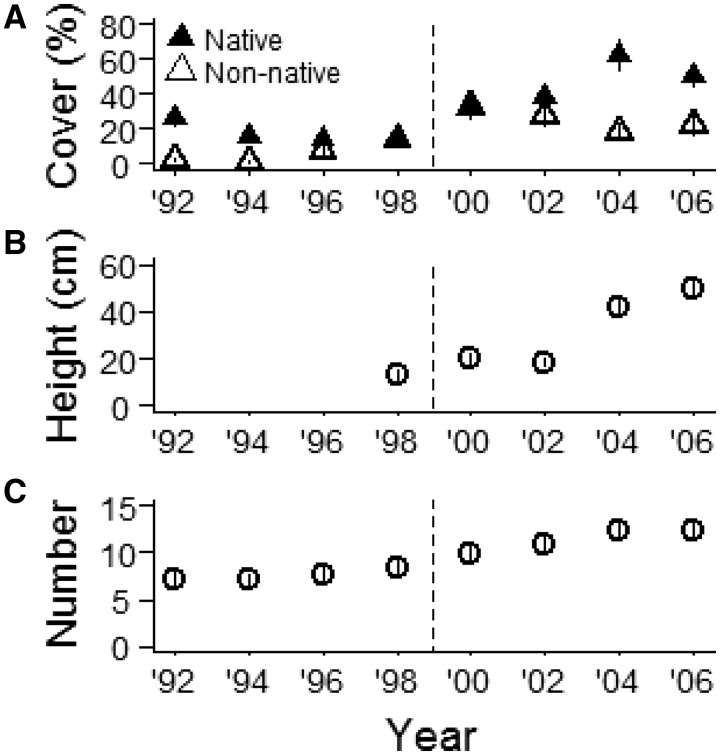
Native and non-native vegetation cover (%) (**A**), vegetation height (**B**) and number of species (**C**) in spring 1992–2006 at Fermilab, IL (*N* = 65–130 one-m^2^ quadrats). Dotted line indicates deer reduction from 24.6 to 2.5 deer per km^2^ in 1998/1999. Data represent means ± 1SE.

**Figure 2.**
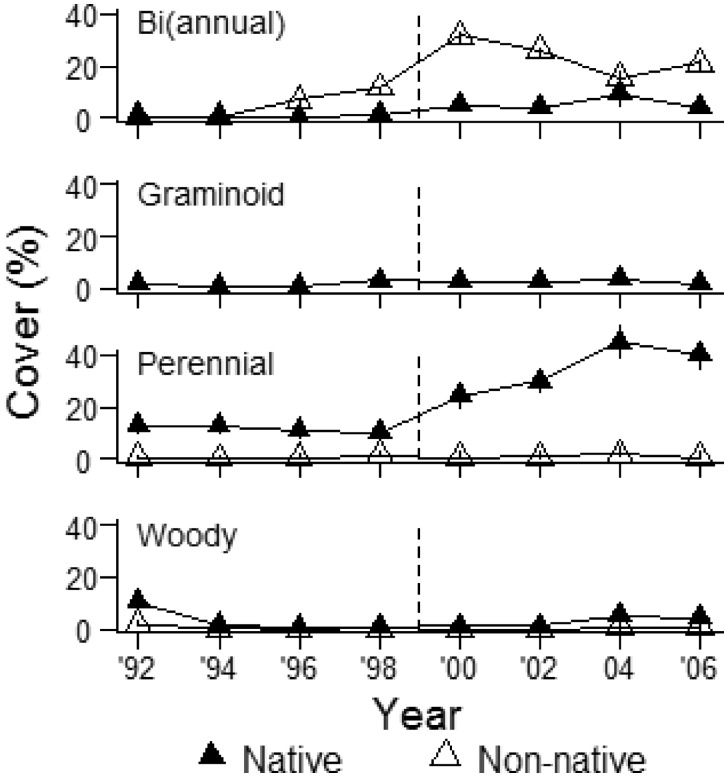
Native and non-native vegetation cover (%) according to life form in spring 1992–2006 at Fermilab, IL (*N* = 65–130 one-m^2^ quadrats). Dotted line indicates deer reduction from 24.6 to 2.5 deer per km^2^ in 1998/1999. Data represent means ± 1SE.

Total cover increased significantly after deer reduction, and cover of native plants increased at a significantly faster rate than cover of non-native plants (significant year × origin interaction; Est ± 1SE: 4.8 ± 2.3, *t* = 2.07, *χ*^2^ = 4.3, df = 1, *P* = 0.04; Fig. 1A). The majority of native cover was contributed by perennial forbs, which increased significantly after deer reduction (Fig. 2; significant year × origin × deer reduction interaction; Est ± 1SE: 0.005 ± 0.0006, *t* = 6.8, *χ*^2^ = 46.3, df = 1, *P* < 0.001). Cover of non-native perennial forbs did not vary over time. Cover of non-native (bi)annuals was significantly higher than native (bi)annuals. While native (bi)annual cover slightly increased after deer reduction, non-native (bi)annual cover peaked the year after deer reduction and then steadily decreased over time (Fig. 2; significant deer reduction × year interaction; *χ*^2^ = 17.18, df = 1, *P* < 0.001). Over 99 % of non-native (bi)annual cover was contributed by a single species, *A. petiolata*. We only encountered native graminoid species and their cover was similar before and after deer reduction (Fig. 2; *P* > 0.05). Native woody cover was higher than non-native woody cover, and increased after deer reduction while non-native woody cover remained stable (Fig. 2; significant year × origin × deer reduction interaction; Est ± 1SE: 0.004 ± 0.0004, *t* = 9.9, *χ*^2^ = 96.2, df = 1, *P* < 0.001). Vegetation height significantly increased after deer reduction, but we observed a lagged response (Fig. 1B; significant deer reduction × year interaction; *χ*^2^ = 9.9, df = 1, *P* = 0.002).

##### Species richness and diversity indexes

Over the 14-year study period we recorded a total of 153 species (13 graminoids, 21 (bi)annuals, 2 ferns, 82 perennial forbs, and 35 woody species), with 77–102 species recorded/year. The majority of species (86 %) were native. Species richness from 1992 to 2006 averaged 9.1 ± 0.15 species per 1 m^2^ quadrat and significantly increased from an average of 7.0 species per quadrat in 1992 to 12.2 species per quadrat in 2006 (+74 %; Fig. 1C). We found a significant effect of deer reduction and a significant interaction between deer reduction and study year (Est ± 1SE: 0.04 ± 0.015, *t* = 2.67, *χ*^2^ = 7.1, df = 1, *P* = 0.008).

Diversity (Shannon index) per quadrat significantly increased from 1.8 ± 0.06 in 1992 to 2.4 ± 0.06 in 2006 and was significantly higher after deer reduction (significant deer reduction × year interaction; *χ*^2^ = 6.9, df = 1, *P* = 0.008). Similarly evenness (Pielou’s index) significantly increased from 0.87 ± 0.02 in 1992 to 0.95 ± 0.02 in 2006 and was significantly higher after deer reduction (significant deer reduction effect; Est ± 1SE: 0.07 ± 0.01, *t* = 7.04, *χ*^2^ = 20, df = 1, *P* < 0.001).

##### Community composition

**Figure 3.**
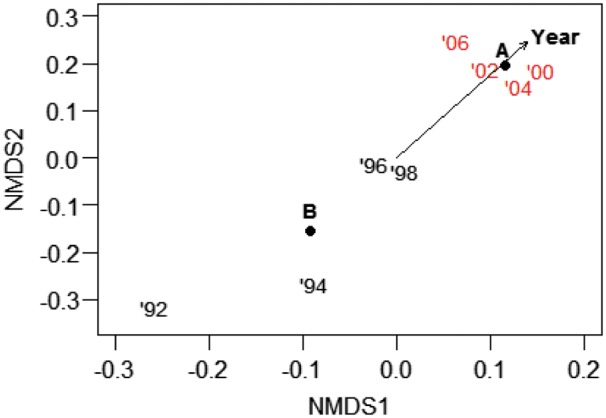
Non-metric multidimensional scaling (NMDS) of the understory vegetation layer in spring 1992–2006 at Fermilab, IL (N = 65-130 one-m^2^ quadrats). Red year numbers indicate monitoring conducted after deer reduction from 24.6 to 2.5 deer/km^2^ in 1998/9. Year, a continuous factor, is represented by a vector (arrow) and deer reduction, a categorical factor indicating before (B) or after (A) the deer cull, by its compositional centroid. Points are jittered to allow visualization.

PerMANOVA results indicated that species composition differed according to study year (*F*_1,884_ = 10.21, *R*^2^ = 0.05; *P* = 0.01), deer reduction (*F*_1,884_ = 56.18, *R*^2^ = 0.05; *P* = 0.01) and the interaction between year and deer reduction (*F*_1,884_=17.3, *R*^2^=0.02; *P*=0.01). Study factors, although significant, explained a small percentage of the variation (indicated by *R*^2^ values). NMDS ordination indicated a difference in vegetation composition before and after deer reduction (Fig. 3). Both study year and deer reduction had a significant goodness of fit (*R*^2^=0.23, *P* < 0.001 and *R*^2^=0.1, *P* < 0.001, respectively). Changes were driven by the six most abundant species: non-native *A. petiolata* and five native early-successional perennial forbs (*Circaea lutetiana*, *Geum canadense*, *Laportea canadense*, *Polygonum virginianum* and *Viola sororia*). Collectively, these six species contributed over 50 % of total cover following deer reduction.

#### Individual species

**Figure 4.**
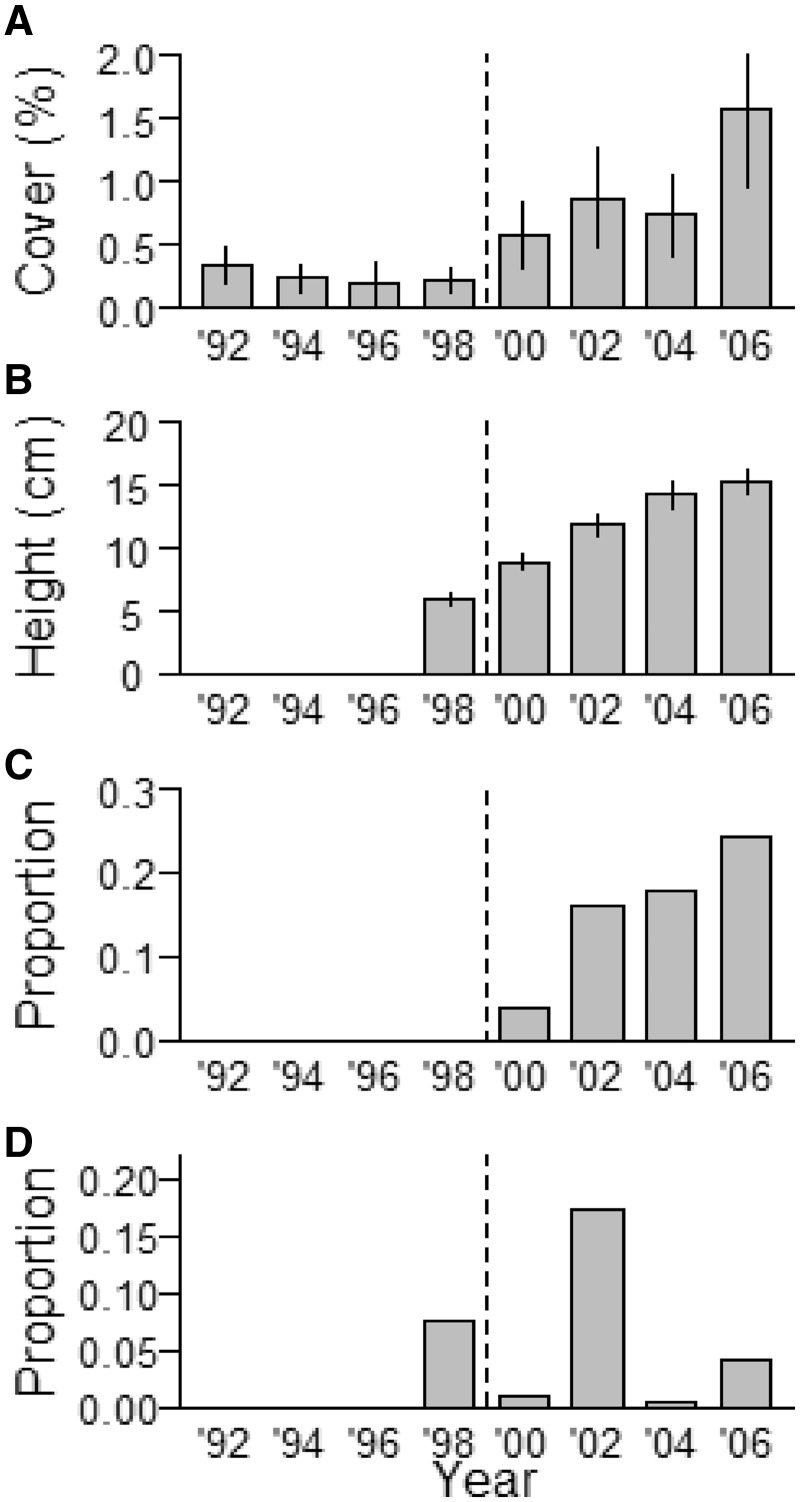
*Trillium recurvatum* percent cover (**A**), height (**B**) (cm; mean ± 1 SE), proportion of flowering individuals (**C**) and proportion of browsed individuals (**D**) in spring 1998–2006 at Fermilab, IL (*N* = 121–128 one-m^2^ quadrats). No individuals flowered in 1998 (**C**). Dotted line indicates deer reduction from 24.6 to 2.5 deer per km^2^ treatment in 1998/1999. We measured cover from 1992 on and other parameters from 1998.

*Trillium recurvatum* cover significantly increased (Est ± 1SE: 0.02 ± 0.007, *t* = 3.3, *χ*^2^=8.4, df = 1, *P* = 0.004) after deer reduction from 0.25 % (average 1992–1998) to 0.93 % (average 2000–2006), reaching maximum cover in 2006 (1.6 %; Fig. 4A). Frequency of quadrats with *T. recurvatum* increased from 26.4 % in 1992 to 33.6 % in 2006, but the increase was not significant (*z* = 1.5, *P* = 0.13). We measured a total of 1142 *T. recurvatum* individual stems over an 8-year period (1998–2006). After deer reduction in 1998/1999, stems became significantly taller (Fig. 4B, Est ± 1SE: −7.9 ± 3.5, *t*=−2.2, *χ*^2^=4.5, df = 1, *P* = 0.03) and had significantly higher flowering probability (Fig. 4C; Est ± 1SE: 0.4 ± 0.5, *z* = 7.33, *P* < 0.001). The proportion of browsed individuals varied dramatically from year to year, but we found no significant effect of deer reduction (Fig. 4D;*z* = 0.75, *P* = 0.45).

### West Point

#### Community

##### Cover and vegetation height

Total cover (averaged across all plots and treatments) significantly increased from 46 % (2009) to 51 % (2012; year effect ± SE: 0.001 ± 0.0004; *χ*^2^=6.88; df = 1; *P*=0.008), but did not differ between open and fenced plots, or between sites with high and low earthworm density. Total cover was significantly higher at sites dominated by non-native vegetation than at sites dominated by native vegetation (site invasion effect ± SE: −0.032 ± 0.01; *χ*^2^=7.2; df = 1, *P* = 0.007).

**Figure 5.**
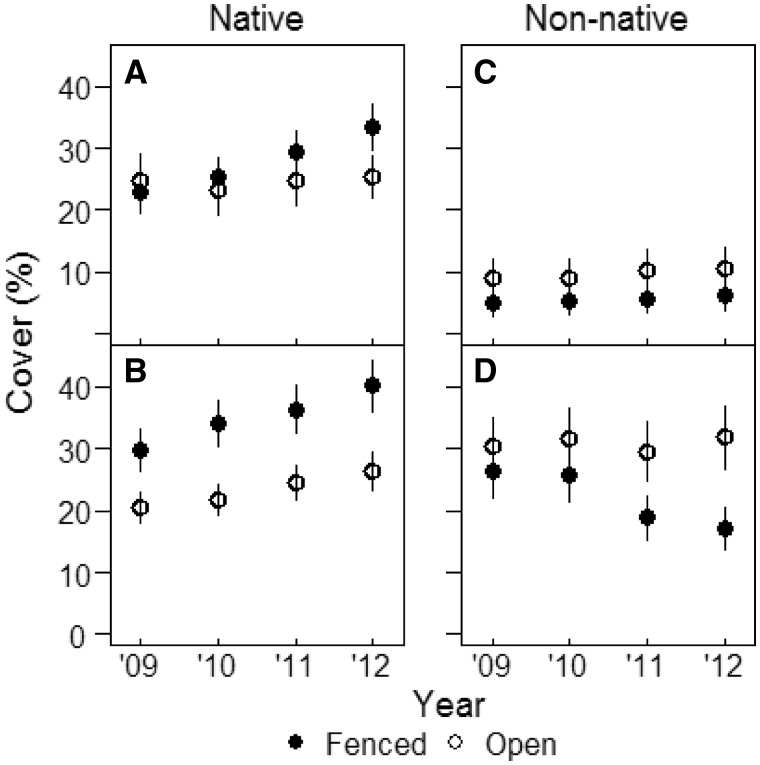
Native (left) and non-native (right) vegetation cover (%) in open and fenced plots (*N* = 10 one-m^2^ quadrats per plot) at sites with low (**A** and **C**) and high (**B** and **D**) earthworm density at West Point, NY from 2009 to 2012. Data represent means ± 1SE (*N* = 4 and 8 sites with low and high earthworm density, respectively).

Proportion of cover contributed by native vs non-native plant species shifted over the study period, influenced by both fencing and non-native earthworm abundance. Native plant species cover significantly increased from 2009 to 2012, whereas non-native plant species cover remained stable (significant origin × year interaction; Table 1). Five years after fencing, native cover was significantly higher in fenced than open plots whereas non-native cover was significantly higher in open than fenced plots (significant origin × fencing interaction; Table 1). Non-native cover was significantly higher at high earthworm density sites than at low earthworm density sites, but native cover was similar between low and high earthworm sites (significant origin × earthworm interaction; Fig. 5; Table 1).

**Figure 6.**
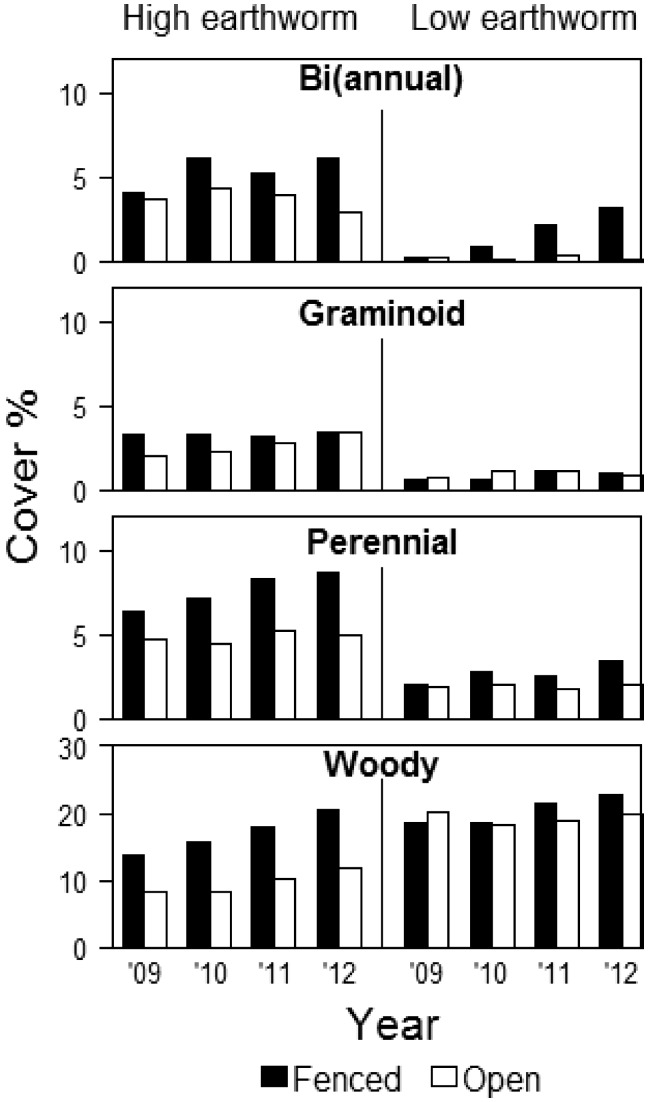
Native vegetation cover (%) according to life form and earthworm density (high or low) in open and fenced plots (*N* = 10 one-m^2^ quadrats per plot) in May–July 2009–2012 at 12 sites at West Point, NY. Data represent means.

Analyses of native cover by life form indicated significant interactions between year, fencing, plant species origin and earthworm density (Table 2). Over the 4 years, averaged across all plots and treatments, native (bi)annual cover (3.31 %) was significantly higher than non-native (bi)annual cover (1.02 %), and native (bi)annual cover tended to be higher at high earthworm sites (3.02 %) than at low earthworm sites (0.50 %; Fig. 6). Native graminoid cover (2 % averaged across all years and treatments) was significantly lower than non-native graminoid cover (9 % on average per quadrat). Both native and non-native graminoid cover were significantly higher at high earthworm density sites than at low earthworm density sites (Fig. 6). We found significant interactions between all study factors (Table 2). Over time, native graminoid cover remained stable in open and fenced plots, whereas non-native graminoid cover decreased in fenced plots (from 9 to 3 %).

**Table 2.** Model results for the effects of study year (Y), plant species origin (O; native, non-native), earthworm density (E; low, high) and fencing (F; open, fenced) on vegetation cover (%) according to life form at 12 sites at West Point, NY from 2009 to 2012. We ran independent linear mixed models for each life form. All models included site, plot within site and quadrat within plot as random factors. Non-significant study factors were dropped from selected models and not included in the table.

Factor		Intercept	Year	Origin (non-native)	Earthworm (low)	Fencing (open)	O × Y	O × E	O × F	Y × F
(Bi)Annuals										
	Est*	0.006		−0.002						
	SE	0.002		0.001						
	*χ*^2^**			15.16						
	*P*			<0.001						
Graminoids										
	Est	3.85	−0.44	9.47	−2.12	−2.34	−1.36	−10.91	5.61	1.29
	SE	3.87	0.51	1.36	6.36	2.15	0.59	1.39	1.31	0.59
	*χ*^2^						5.37	82.5	121.47	4.85
	*P*						0.02	<0.001	<0.001	0.03
Perennials										
	Est	0.022		−0.021	−0.01	−0.004		0.009	0.004	
	SE	0.002		0.001	0.002	0.001		0.001	0.001	
	*χ*^2^							109.51	24.82	
	*P*							<0.001	<0.001	
Woody										
	Est	0.027	0.001	−0.015	0.011	−0.006		−0.013	0.009	
	SE	0.005	0.0005	0.002	0.008	0.002		0.002	0.002	
	*χ*^2^		5.22					34.29	18.61	
	*P*		0.02					<0.001	<0.001	

Est Estimate; SE estimate standard error.

* Estimates and standard errors are reported from the model fitted with restricted maximum likelihood.

** Chi-squared statistics and *P*-values are from likelihood ratio tests with each parameter removed from the maximum likelihood-based model, with all other parameters retained. It was not possible to test the significance of all terms because of higher order interactions. All chi-square tests have 1 degree of freedom.

Perennial native cover (5 % averaged across all years and treatments) was significantly higher than non-native perennial cover (0.12 %) and both native and non-native perennials had higher cover at sites with high earthworm density and in fenced than open plots (Fig. 6; Table 2).

Overall, native woody cover was significantly higher than non-native woody cover (15 and 10 % averaged across all years and treatments, respectively). We found a significant interaction between plant species origin and earthworm density (Table 2): native woody cover was significantly higher at low earthworm density sites than at high earthworm sites, whereas non-native woody cover was not significantly correlated with earthworm density. We also found a significant interaction between origin and fencing: native woody cover was significantly higher in fenced than open plots (Fig. 6), whereas non-native woody cover was significantly higher in open than in fenced plots.

Separate analyses for May 2016 (*N* = 10 sites, two sites excluded from analysis due to fence damage) showed similar cover at low and high earthworm density sites and between open and fenced plots (*P* > 0.05), but a significant effect of site invasion (*F*_1,14_=5.33, *P* = 0.04). Total cover was significantly higher at the five sites dominated by non-native vegetation (42 ± 17.1 %; mean ± 1SE; *N* = 5 sites; values reflect mean of median cover per plot) than at the five sites dominated by native vegetation (23 ± 3.9 %). We found no significant effect of fencing or earthworm density on vegetation height (*P* > 0.5).

##### Species richness and diversity indexes

Over the 5-year study period we recorded a total of 192 species (36 graminoids, 17 (bi)annuals, 8 ferns, 78 perennial forbs and 53 woody species). The majority of species (92 %) were native. Species richness was not affected by fencing, earthworm density or site invasion, but significantly decreased over the study period at both the 1 m^2^ quadrat scale and 900 m^2^ plot scale. Species richness over 2009–2012 averaged 7.3 ± 0.17 species per 1 m^2^ quadrat and significantly decreased from 7.82 species on average in 2009 to 7.00 species in 2012 (year effect ± SE: −0.29 ± 0.09; *χ*^2^=9.1; df = 1, *P* < 0.01), and significantly decreased from 30.00 ± 3.34 species per plot in 2009 to 27.45 ± 2.94 species in 2012 (year effect ± SE: −0.85 ± 0.25; *χ*^2^=10.47; df = 1, *P* < 0.001).

Diversity (Shannon index) per open or fenced plot slightly but significantly increased over the study period from 1.70 ± 0.17 in 2009 to 1.80 ± 0.16 in 2012 (year effect ± SE: −0.035 ± 0.01; *χ*^2^=5.07; df = 1, *P* = 0.02). Similarly evenness (Pielou’s index) significantly increased from 0.50 ± 0.04 in 2009 to 0.54 ± 0.04 in 2012 (year effect ± SE: 0.015 ± 0.003; *χ*^2^=6.6; df = 1, *P* = 0.01). Neither diversity nor evenness were correlated with fencing, earthworm density or site invasion.

Species richness in May 2016 averaged 24.5 ± 2.92 species per plot, Shannon Diversity Index averaged 3.0 ± 0.15 and Pielou’s evenness averaged 0.95 ± 0.01. Fencing and earthworm density had no effect on either measure.

##### Community composition

PerMANOVA results indicated that species composition differed according to earthworm density (*F*_1,954_=39.83, *R*^2^=0.04; *P* < 0.01) and fencing (*F*_1,954_=3.39, *R*^2^=0.003; *P* = 0.01), with a significant interaction between earthworms and fencing (*F*_1,954_=2.85, *R*^2^=0.002; *P* = 0.01). Study factors, although significant, explained a small percentage of the variation (indicated by *R*^2^ values). We found no significant year effect, and no interactions between study year and fencing or earthworms on plant species composition. The interaction between earthworm density and fencing did not change over time and therefore we do not attribute it to our fencing treatment.

**Figure 7.**
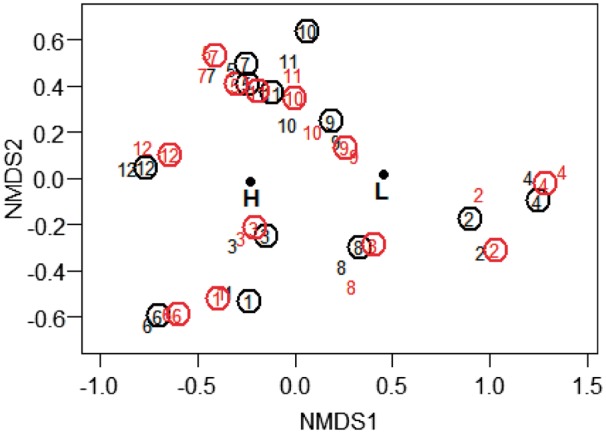
NMDS of the understory vegetation layer in May and July 2009 and 2012 in open and fenced plots at 12 sites at West Point NY (*N* = 10 permanent monitoring quadrats per plot). Numbers indicate study sites. Black and red numbers indicate sampling year (2009 and 2012, respectively) and circled numbers represent fenced plots. Earthworm density, a categorical factor indicating low (L) or high (H) earthworm density is represented by its compositional centroid. Points are jittered to allow visualization.

NMDS ordination showed no difference in understory plant community composition between open and fenced plots (*R*^2^=0.001, *P*=0.5), either at the beginning (2009) or end (2012) of the study period (*R*^2^=0.0001, *P*=0.9; Fig. 7). Earthworm density, on the other hand, had a significant goodness of fit (*R*^2^=0.18, *P* < 0.001) with low- and high-density earthworm sites separating along the first axis. Sites grouped according to dominant plant species. For example, sites dominated by *M. vimineum* (sites 3 and 12) and by *B. thunbergii* (sites 1 and 6) formed clear separate clusters. On the other hand, *A. petiolata* sites (sites 5 and 7) clustered with sites dominated by native vegetation.

#### Individual species

**Figure 8.**
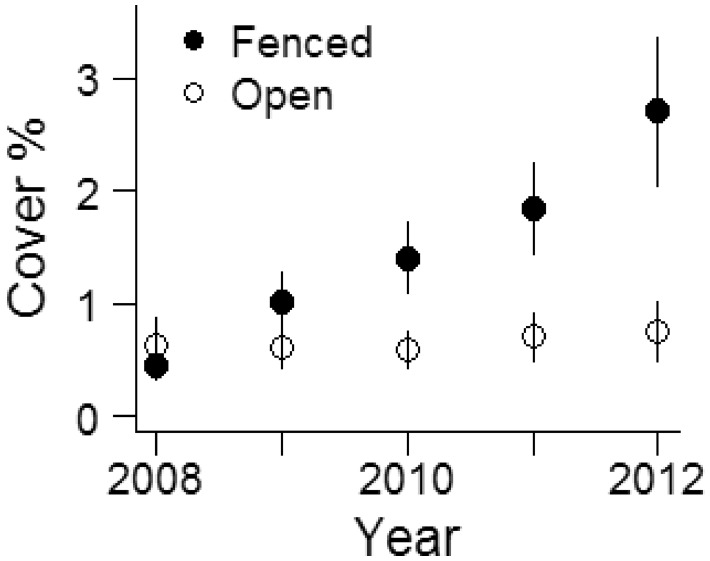
*Eurybia divaricata* cover (%) in open and fenced plots (*N* = 10 one-m^2^ quadrats per plot) in July 2008–2012 at six sites at West Point, NY. Only sites where *E. divaricata* was present were included in the study. Data are means ± 1SE.

**Figure 9.**
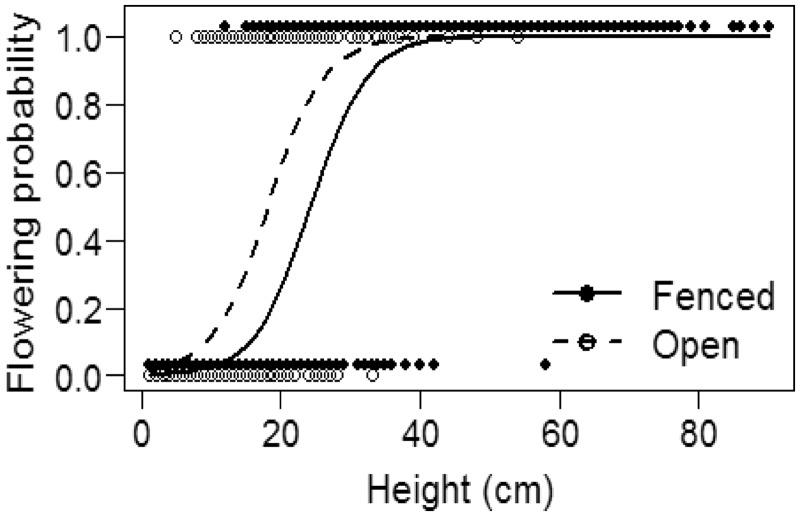
Flowering probability of *Eurybia divaricata* as a function of stem height in open and fenced plots at six sites at West Point, NY in June 2012. Lines depict model predictions.

Between 2008 and 2012, *E. divaricata* mean cover in permanent vegetation quadrats significantly increased in all fenced plots, and remained relatively constant in all open plots (significant year × fencing interaction; interaction effect ± SE: −0.016 ± 0.05; *χ*^2^=10.1, df = 1, *P* = 0.001; Fig. 8). Frequency remained relatively constant in all plots during the same period (data not shown). We measured 942 individual *E. divaricata* stems, distributed across six sites (only sites where the species was present were included in the study). Plants in the fenced plots were significantly taller (Est ± 1SE: 20.27 ± 3.5, *t*=−5.7, *χ*^2^=17.6, df = 1, *P* < 0.001) and more likely to flower (Est ± 1SE: 1.5 ± 0.4, *z* = 3.7, *P* < 0.001). Interestingly, fertile plants in open plots were significantly shorter than fertile plants in fenced plots (Fig. 9).

**Figure 10.**
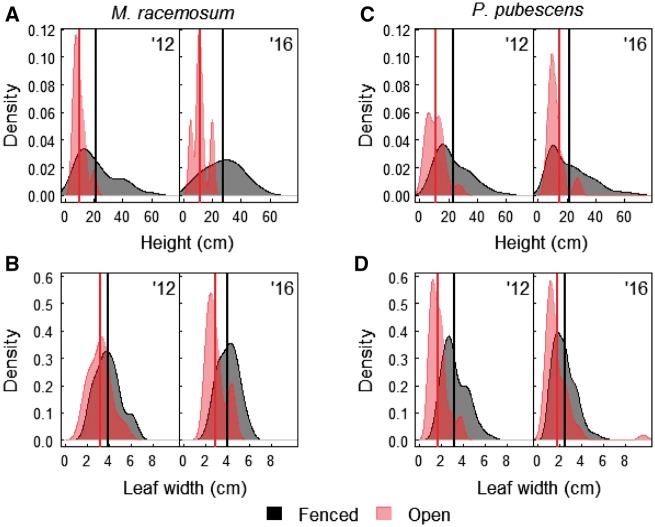
Probability distribution of stem height (cm) and leaf width (cm) of *Maianthemum racemosum* (**A** and **B**) and *Polygonatum pubescens* (**C** and **D**) in open and fenced plots at two sites at West Point, NY in June 2012 and 2016. Fences were erected in 2008. Black and red solid lines represent means at fenced and open plots, respectively.

*Maianthemum racemosum* was uncommon at both sites in both years. In total, we found and measured 50 *M. racemosum* in 2012 and 24 in 2016, of which 12 (24 %) and 5 (21 %) were in open plots in 2012 and 2016, respectively. Stem height (Est ± 1SE: −0.74 ± 0.14, *t*=−5.2, *χ*^2^=7.1, df = 1, *P* = 0.01) and leaf width (Est ± 1SE: −0.27 ± 0.06, *t*=4.33, *χ*^2^=6.4, df = 1, P = 0.01) were significantly higher in fenced (mean ± 1SE; height: 23.69 ± 1.8 cm; leaf width: 3.96 ± 0.14 cm) than open plots (height: 10.4 ± 1.1 cm; leaf width: 3.11 ± 0.23 cm; Fig. 10). We found no flowering *M. racemosum* in open plots at either site, but 32 % and 16 % of plants flowered in fenced plots at sites 5 and 8, respectively. Flowering plants were significantly taller (Est ± 1SE: 0.75 ± 0.15, *t*=5.1, *χ*^2^=21.9, df = 1, *P* < 0.001) than non-flowering plants at both sites.

We measured 109 *P. pubescens* stems in 2012 and 274 in 2016. Similar to *M. racemosum* we recorded fewer stems in open (45 and 51 in 2012 and 2016, respectively) compared with fenced plots (64 and 223 in 2012 and 2016, respectively) despite the larger sampling area in open plots. We found a significant positive effect of fencing on stem height (22.32 ± 0.82 cm in fenced vs 13 ± 1.62 cm in open; Est ± 1SE: −0.56 ± 0.12, *t*=−4.67, *χ*^2^=6.9, df = 1, *P* = 0.01). In contrast, leaf width did not differ between open and fenced plots (2.6 ± 0.08 cm in fenced vs 1.7 ± 0.12 cm in open; *P* > 0.05; Fig. 10). At site 5, plants had a significantly higher probability of flowering in fenced than open plots (Est ± 1SE: −1.19 ± 0.43, *z* = 2.73, *P* = 0.006). At site 8, we recorded only one flowering individual in the open plot in 2012 and no flowering individuals in 2016, preventing formal analysis. Nevertheless, 41 % and 43 % of plants in the fenced plot flowered in 2012 and 2016, respectively. Flowering plants were significantly taller than non-flowering plants (Est ± 1SE: 0.99 ± 0.05, *t* = 20.69, *χ*^2^=287.9, df = 1, *P* < 0.001). 

## Discussion

We used two commonly employed methods, deer culling and fencing, to assess whether significant deer population reduction or even deer browse cessation has potential to allow for forest understory recovery. Our results indicate that based on how we define recovery, we can either accept or reject our first hypothesis. If we define recovery as increased abundance, however subtle, of native understory forest plant species, then we can accept our first hypothesis that reduced deer browse pressure can result in recovery of understory communities. At both Fermilab and West Point, cover of native species increased and cover of non-native species decreased in response to reduced deer abundance. At Fermilab recovery was dramatic with cover and height increasing rapidly, but this response was driven by explosive growth of early successional species already present in the community. At West Point change was more nuanced as total cover (native and non-native combined) was similar inside and outside fenced plots over the study period. At both locations, species present before deer reduction remained present afterwards, and few new species appeared.

However, if we define recovery more specifically, for example as increased cover and diversity of a broad array of plant species typical of the forest understory and including deer-favoured and late successional species, then we must reject our first hypothesis. Culling or fencing alone, despite greatly reducing deer browse pressure, was unable to bring back preferred deer browse species to a point that their presence was detectable at the community monitoring level—not even over a decadal time period. Limited recovery of species highly favoured by deer occurred (Hypothesis 2) but was only detectable when monitoring individual species. And this recovery occurred only for species that remained present within the community. This is, in part, explained by the present-day infrequent occurrence, particularly of reproductive individuals, of these deer-favoured species over much of their range (Ludwig and Conklin 1992; Wang and Mopper 2008; Knight *et al.* 2009a). Our results corroborate others in describing a lack of a vegetative response to fencing or substantial deer reductions, particularly for those species that appear threatened by excessive deer browse (Royo *et al.* 2010; Goetsch *et al.* 2011; Pendergast *et al.* 2016). Without further intervention, even after complete exclusion of deer, forests may remain in a depauperate stable state (Augustine *et al.* 1998; Nuttle *et al.* 2014) that developed over many decades in response to high deer populations.

Our first hypothesis was articulated in anticipation of a demographic response of those species that have suffered high browse intensity over extended time periods. Neither at West Point, nor at Fermilab, did we have access to detailed community composition data from before deer population increases, thus we are not fully able to “ground truth” the response, or better lack thereof, of the understory vegetation community. But in both regions floras and early forest community descriptions imply substantially different plant community compositions (Pepoon 1927; Raup 1938; Swink and Wilhelm 1979), and to a major extent the deer browse sensitive species are those that are now missing in the local forests or occur at greatly suppressed abundances (Ludwig and Conklin 1992; Werier and Barbour 2012).

An obvious question then is whether it was in fact the increase in deer populations, rather than abundance of non-native plants and non-native earthworms, that is responsible for the now widespread depauperate plant communities at our sites? Such negative effects of high deer populations on local plant and animal diversity, abundance, and cover have been described for many other locations and regions using long-term datasets (Côté *et al.* 2004; Wiegmann and Waller 2006; Rogers *et al.* 2009; Goetsch *et al.* 2011; Martin *et al.* 2011; Frerker *et al.* 2014; Chips *et al.* 2015) and our sites have likely undergone similar “sorting” processes. While we lack detailed historic records from our study locations, we documented that protecting individuals of preferred browse species such as *T. recurvatum*, *E. divaricata*, *M. racemosum* and *P. pubescens* from deer browse by fencing, or lowering deer browse pressure by culling, had beneficial effects on growth and reproductive output (Figs. 4 and 8–10), two important factors that may ultimately contribute to positive population growth rates (Wang and Mopper 2008; Knight *et al.* 2009a; Kalisz *et al.* 2014). This benefit accrued despite the remaining potential threats of biotic influences (invasive plants and introduced earthworms) or other regional factors such as climate change, changes in nutrient deposition or previous land use history that were not affected by deer culling or fencing. Thus, we find support for our third hypothesis. Together, this provides clear and compelling evidence that deer are the responsible driver (MacDougall and Turkington 2005) for demise of native species, and that the rise of at least the three non-native invasive plant species we investigated is but a symptom of other underlying stressors (see also Blossey et al, this issue). Thus, these three non-native species can be considered passengers rather than drivers of change in plant community composition (MacDougall and Turkington 2005; Dávalos *et al.* 2015b).

Our results further demonstrate that the effect of deer browse on vegetation is influenced by earthworm density, and deer browse and earthworms interact to influence growth of different plant life forms (Figs. 5 and 6). We are unable to assess how plant invasion and deer browse intensity have interacted historically to facilitate changes in native or introduced plant abundance. But earthworm abundance itself is a function of deer presence, deer exclusion results in declines of earthworms over time (Dávalos *et al.* 2015c), and earthworms also decline as forests age (Simmons *et al.* 2015) suggesting a complicated web of interactions that we are only slowly beginning to understand (Dobson and Blossey 2015; Craven *et al.* 2016). At West Point, both native and non-native vegetation composition was strongly affected by earthworm density at the beginning of our investigations (Fig. 5), which in turn may have been influenced by site-specific factors such as soil pH, moisture or invasion history (Dávalos *et al.* 2015c; Paudel *et al.* 2016) and both native and introduced species responded more strongly to deer exclusion in areas with high earthworm density.

Our work confirms the work by others that non-native vegetation cover, abundance, growth and population growth rates decline when deer browse pressure is reduced or even eliminated (Kalisz *et al.* 2014; Dávalos *et al.* 2015b) although see Averill *et al.* (this issue). We suggest that non-native plants, at least the three species in our study (*A. petiolata, B. thunbergii, M. vimineum*) are indicators of earthworm invasion and high deer abundance, and that these two factors pose greater threats, singly and in combination, to native vegetation than do non-native plants.

There are strong legacy effects of long-term high deer abundance and non-native earthworm abundance (Dávalos *et al.* 2015c) that impact recovery of vegetation and community composition even after deer reduction. Under continuous intense browsing, forest understory vegetation can be nearly eliminated, leaving an empty forest floor (Goetsch *et al.* 2011). Once highly favoured and/or uncommon species are eliminated or greatly reduced, the trajectory of community recovery after deer reduction will shift, and the resultant community may have low similarity to the previous community. Deer legacy effects include altered tree recruitment with resulting ripple effects on invertebrate and bird communities (Nuttle *et al.* 2011), and altered herbaceous forest layer that does not have a height refuge to escape deer browse (Nuttle *et al.* 2014; Chips *et al.* 2015). Deer and introduced earthworm abundance interact (Dávalos *et al.* 2015c) and may affect soil microbial community ability to facilitate or suppress germination and growing conditions for other native species through plant-soil feedbacks (Kardol *et al.* 2007, 2014). These indirect yet important non-consumptive deer effects have been overlooked until recently and they include demographic effects (Heckel *et al.* 2010; Dávalos *et al.* 2014) as well as evolutionary effects on plant height, palatability, or height needed to initiate reproductive efforts (Martin *et al.* 2015; Prendeville *et al.* 2015; Holeski *et al.* 2016).

Our work indicates that recovery following deer reduction is dependent upon the species that are already present (as plants or in seedbanks), and species that seed in (mostly wind or animal dispersed). Rescue from long-lived existing seed banks, as is typical for many wetland plant species (van der Valk 1981), does not represent an important demographic response available for many forest understory species (Nuzzo *et al.* 2015). Forest seed banks are typically dominated by early successional species dependent on high light conditions and disturbances for germination and growth, with a noticeable absence of perennial monocots and long-lived herbaceous species with low reproductive effort and extended germination requirements (Matlack and Good 1990; Hopfensperger 2007; Bossuyt and Honnay 2008; Schmidt *et al.* 2009; Esmailzadeh *et al.* 2011; Levine *et al.* 2012; Beauchamp *et al.* 2013). Furthermore, deer browsing affects seedbank composition and reduces germinant species diversity and richness, and total number of germinants (DiTommaso *et al.* 2014). Even if propagules arrive (as seeds or transplants), deer abundance may influence germination and growth (Dávalos *et al.* 2015a), and plant–plant competitive effect may also hinder establishment. In addition, non-native earthworms influence seedbank composition, seedling emergence and seedling survival (Willems and Huijsmans 1994; Regnier *et al.* 2008; Eisenhauer *et al.* 2010; Clause *et al.* 2015; Dobson and Blossey 2015; Nuzzo *et al.* 2015). Maintaining presence and abundance of large reproductive individuals will be paramount for conservation of many deer browse preferred herbaceous species (Wang and Mopper 2008; Knight *et al.* 2009a; Dávalos *et al.* 2014; Kalisz *et al.* 2014).

We expected that using typical plant community monitoring in 1 m^2^ quadrats would allow us to capture (1) changes in plant community structure associated with reduced deer browse pressure, and (2) the recovery of species highly favoured by deer. Our data show that we can accept the first expectation (albeit the effect was very limited and we found it only at Fermilab for early successional species) but reject the second. At Fermilab, understory composition prior to the cull changed gradually, slightly differing year after year (Fig. 3), whereas after the cull vegetation composition did not differ among years, emphasizing the rapid dominance of a few early successional species, potentially suggesting a continued lagged or legacy response that was not remedied by a greatly reduced deer population. At West Point we did not capture a community response to fencing at any of the 12 sites (Fig. 7). At our sites monitoring at the community level was unable to capture the subtle positive effects of deer browse reduction on individual species, at least over the time frames used in our studies. Three of the four species that we monitored (*M. racemosum*, *P. pubescens* and *T. recurvatum*) require 2 years to emerge from seed, and 7–10 years to produce a first flower (Baskin and Baskin 1992; Case and Case 1997; Cullina 2000). To effectively capture a demographic success (initial colonization and a population increase) in these and similar slow-growing species, it may be necessary to monitor for a decade or longer using traditional permanent 1 m^2^ quadrats.

We are not the first to document the limitations of plant community monitoring: a recent meta-analysis reported on inconsistencies in outcomes using this approach (Habeck and Schultz 2015). Interestingly, community monitoring is able to capture the initial “erosion” of diversity when deer colonize previously deer free islands or refugia (Côté *et al.* 2004; Mudrak *et al.* 2009; Martin *et al.* 2011), but is less effective at capturing recovery after deer reduction. Furthermore, community monitoring can be a powerful tool but requires substantial expertise to identify all plants to species, and is time consuming and hence expensive. Our results show that it also may not be the best approach to capture the initial response of vegetation to deer reductions. Monitoring individual deer-favoured plant species is both easier and less time-consuming assuming they still exist in local forests albeit at greatly reduced abundances. The response of individuals of deer favoured plant species clearly demonstrates that abundant deer herbivory reduces performance of these species, potentially for decades.

The differences in the height needed to initiate flowering between plants in open and fenced plots further suggests that chronic deer herbivory may over time favour plants with lower stature in areas where they are exposed to high deer herbivory. How these eco–evolutionary interactions play out over the long term, and how they could affect plant demography remains to be investigated. It is possible that decades of intensive deer browse may result in individuals of the remaining plant species that through phenotypic plasticity are reasonably well able to cope with deer browse but are poor performers or contributors to population growth rates due to reduced reproductive activity and stature. This may negatively affect the long-term viability of populations exposed to chronic deer herbivory.

Legacy effects of prolonged high deer browse intensity often result in understories that show little response to deer reductions, especially by vulnerable species (Royo *et al.* 2010; Nuttle *et al.* 2014; Pendergast *et al.* 2016). Expectations for rapid wholesale beneficial changes or return of species that have suffered from excessive deer browse pressure for decades and that have retreated to small refugia or have been lost from entire forests are not met by our evidence or results by others (Carson *et al.* 2005; Comisky *et al.* 2005; Ripple and Beschta 2005; Krueger and Peterson 2006; Habeck and Schultz 2015). Many of the currently remaining small populations may face an extinction debt (Vellend *et al.* 2006; Rogers *et al.* 2009) and we may lose them unless appropriate conservation measures are implemented. This may include not only greatly reduced deer abundance to provide safe spaces for recruitment, but also active restoration if we want to retain these species as part of our biological communities and heritage.

Recognizing the limitations of approaches and metrics (such as plant community monitoring) to measure incremental long-term plant responses to changes in deer populations and deer browse pressure is an important part of management of forest understory communities. This includes the recognition that forest recovery needs to be appropriately defined and “simple” suggestions such as “increased diversity” or “increased native cover” as an outcome of deer management may be insufficient to safeguard or recover browse sensitive species that have dramatically declined over the past decades. Defining recovery through growth, ability to reproduce and positive population growth rates for individual species may be much more appropriate.

Our assessment of plant individuals shows that browse sensitive individuals (if still present in the areas of interest) can be utilized to assess incremental initial success, or lack thereof, of deer management (culling, fencing, hunting, sterilization or no management). Where these species are absent, planting of indicator species may be warranted (see Blossey *et al*. 2017).

## Conclusions

Deer culling or fencing results in reduced non-native plant cover and increased native plant cover, but little change in plant community composition (although a few early successional native forbs can increase rapidly under reduced deer browse). We found that deer herbivory interacts with impacts of introduced earthworms in shaping plant communities, especially growth of different life forms. We also found that non-native plant species (at least the three in this study) had minimal effect on plant community composition. Rather, they are indicators of other stressors, especially presence of non-native earthworms and deer abundance.

Plant community monitoring fails to capture initial and subtle effects of reduced or even cessation of deer browse on browse sensitive species, especially when these species are rare or are not present in standardized monitoring quadrats, as occurred in our study. Measuring responses of individual plants (growth, flowering and reproductive success) provides a more sensitive and powerful assessment of forest understory responses to deer management.

Land managers need to appropriately define recovery goals of deer management and develop suitable metrics. Defining recovery through growth, ability to reproduce and positive population growth rates for individual species is more appropriate than simply stating ‘increased species diversity’ or ‘increased native cover’. Reducing deer populations may provide safe spaces for recovery of deer sensitive species, but active restoration may be required if we want to retain these species as part of our ecosystems.

## Sources of Funding

Funding for the Fermilab research was provided by U.S. DOE Fermilab National Environmental Research Park (0perated by Fermi Research Alliance, LLC under Contract No. DE-AC02-07CH11359 with the United States Department of Energy) to V.N. Funding for the West Point research was provided by the Strategic Environmental Research and Development Program (SERDP) of the U.S. Department of Defense (Grant RC-1542 to BB).

## Contributions by the Authors

V.N. conceived the Fermilab study design, and B.B. and V.N. conceived the West Point study design; V.N. collected plant data, and A.D. and V.N. collected earthworm data. A.D. conducted all analyses. B.B. led the writing and all authors participated in writing the manuscript.

## Conflicts of Interest Statement

No conflicts of interest.
